# The *Microcystis*-microbiome interactions: origins of the colonial lifestyle

**DOI:** 10.1093/femsec/fiae035

**Published:** 2024-03-18

**Authors:** Claudia Piccini, Gabriela Martínez de la Escalera, Angel M Segura, Carolina Croci, Carla Kruk

**Affiliations:** Departamento de Microbiología, Centro de Investigación en Ciencias Ambientales, Instituto de Investigaciones Biológicas Clemente Estable. Av. Italia 3318, Montevideo 11600, Uruguay; Departamento de Microbiología, Centro de Investigación en Ciencias Ambientales, Instituto de Investigaciones Biológicas Clemente Estable. Av. Italia 3318, Montevideo 11600, Uruguay; Modelización Estadística de Datos e Inteligencia Artificial, Centro Universitario Regional del Este, Universidad de la República. Ruta nacional Nº9 intersección con ruta Nº15, Uruguay; Departamento de Microbiología, Centro de Investigación en Ciencias Ambientales, Instituto de Investigaciones Biológicas Clemente Estable. Av. Italia 3318, Montevideo 11600, Uruguay; Modelización Estadística de Datos e Inteligencia Artificial, Centro Universitario Regional del Este, Universidad de la República. Ruta nacional Nº9 intersección con ruta Nº15, Uruguay; Sección Limnología, Instituto de Ecología y Ciencias Ambientales, Facultad de Ciencias, Universidad de la República. Iguá 4225, Montevideo 11400, Uruguay

**Keywords:** colonies, EPS, holobiont, microbiome, *Microcystis*, mucilage

## Abstract

Species of the *Microcystis* genus are the most common bloom-forming toxic cyanobacteria worldwide. They belong to a clade of unicellular cyanobacteria whose ability to reach high biomasses during blooms is linked to the formation of colonies. Colonial lifestyle provides several advantages under stressing conditions of light intensity, ultraviolet light, toxic substances and grazing. The progression from a single-celled organism to multicellularity in *Microcystis* has usually been interpreted as individual phenotypic responses of the cyanobacterial cells to the environment. Here, we synthesize current knowledge about *Microcystis* colonial lifestyle and its role in the organism ecology. We then briefly review the available information on *Microcystis* microbiome and propose that changes leading from single cells to colonies are the consequence of specific and tightly regulated signals between the cyanobacterium and its microbiome through a biofilm-like mechanism. The resulting colony is a multi-specific community of interdependent microorganisms.

## Teaser

*Microcystis* is a unicellular genus of Cyanobacteria provoking dense blooms formed by colonies of different sizes, which result from specific interactions between the cyanobacterium and its microbiome through a biofilm-like mechanism.

## Introduction

It has been since a long time ago that microbiologists have noticed that bacteria do not always live as single cells. Many of the known bacterial species have the ability to grow in a multicellular and coordinate way, the biofilms. Bacterial biofilms are defined as aggregates of microbial cells surrounded by a self-produced polymer matrix that can be composed by a single (mono-specific) or several species (multi-specific) living in a collaborative way. Biofilm growth of microorganisms was first defined in medical microbiology, when it was also demonstrated that biofilm-embedded organisms have an increased antimicrobial resistance compared to those growing as planktonic bacteria (Nickel et al. 1985).

The classic conceptual model of biofilm formation involves motile planktonic cells that become attached to a surface in response to a variety of environmental signals (Sauer et al. 2022). Attached cells produce a hydrated matrix of extracellular polysaccharides (EPS), extracellular DNA, proteins and lipids (Flemming and Wingender 2010), changing their structure and functional relationships. After a while, sessile cells arranged in microcolonies from where some cells can escape to return to the planktonic lifestyle and subsequently colonize a new surface (Petrova and Sauer 2016). Although biofilm cells encounter stronger gradients of nutrients and waste products than during planktonic life, they are embedded in a more controllable environment (Stewart and Franklin 2008).

In the case of aquatic cyanobacteria, despite the increasing amount of information regarding their ecology, the biofilm concept is generally associated with benthic species, which form mats in several aquatic ecosystems (Stal 2012). Among the planktonic groups we will focus on *Microcystis* spp., a complex of cyanobacteria from the Chroococcales order that live in freshwater and brackish waters. They form dense blooms in eutrophic ecosystems (Paerl 1988, Huisman et al. 2018) and can be found as single cells or in colonies floating near the surface, with a size spectrum ranging from ca. 4 µm (single cells) to hundreds of microns (large colonies) (Reynolds et al. 1981) that can be detected by naked eye.

Interestingly, *Microcystis* belongs to a phylogenetic group of unicellular cyanobacteria and its ability to form colonies is usually considered as an ecological aggregation strategy to avoid predation or protect from ultraviolet radiation, among others. In this context, colony formation by these organisms has been explained either by cell division (the usual bacterial process to multiply) or cell adhesion (Yang et al. 2008). However, recent genomic evidence suggests that colonies in *Microcystis* result from clonal expansion rather than cell aggregation (Carrascal et al. 2021).

In spite of the amount of information regarding *Microcystis* ecology, colony formation and toxicity, little is known about the biological interactions taking place inside the colony and their role in *Microcystis* biology and evolution. Here, we focus on (i) the characteristics shared by bacterial biofilms and *Microcystis* colonies; (ii) the current knowledge about colony formation process in *Microcystis*; (iii) the evidence on the existence of quorum sensing (QS) in *Microcystis* and; (iv) the information about community composition and function of the colony-associated microbiota. Based on this, we propose that the morphological, functional and microbiome compositional changes occurring from single cells to colonies are consequence of biological and ecological interactions between the cyanobacterium and the heterotrophic bacteria. These specific and carefully regulated interactions are bi-directional and induce the development of a mucilaginous envelope that will host the heterotrophic community through a biofilm-like mechanism. Taking this into account a conceptual model of emergence and decay of these floating multi-specific biofilms of *Microcystis* is presented.

### *Microcystis* blooms and microcystins production

*Microcystis* blooms are composed by a mixture of populations able to produce secondary metabolites called microcystins that are toxic to animals and humans, and by non-toxic populations. It has been shown that high water temperature (between 25 and 30°C) promotes the growth of *Microcystis* populations able to produce microcystin (toxic), while non-toxic populations seem to have less tolerance to variable environmental conditions (Davis et al. 2009, Van de Waal et al. 2011). Therefore, it is very likely that under the current climate warming and worldwide eutrophication scenario a dominance of cyanobacterial blooms containing a higher percentage of toxic *Microcystis* will occur (Paerl and Huisman 2008, Kruk et al. 2023), making it very relevant to understand the biology and ecology of these organisms.

Until now, studies on the ecology of *Microcystis* have focused on the determinants of its growth, potential toxicity and diversity (Dick et al. 2021). More recently, the structure and function of its microbiome and its role in the survival and fitness of the cyanobacterium have started to be included (Jankowiak and Gobler 2020, Schmidt et al. 2020, Carrascal et al. 2021). However, there is no consensus on the mechanisms that determine the production of microcystins, the density and persistence of blooms or the microbiome community structure. In this sense, the evidence from different studies is frequently contradictory, since some works are based on axenic cultures of unicellular forms (hard to find in nature), others on environmental samples and others analyse and compare sequences obtained either from isolates, environmental DNA or enrichments from blooms, making generalizations difficult (Pimentel and Giani 2014, Martin et al. 2020, Zhou et al. 2020, Yang et al. 2022, Dai et al. 2023, Yin et al. 2024). Another possible explanation for the contradictions is that the factors driving bloom formation may be uncoupled from those driving toxicity, perhaps due to complex regulation pathways associated not only to the cyanobacterium, but also to its heterotrophic partners.

### Similarities between *Microcystis* colonies and biofilms

In biofilms, attached cells produce a hydrated matrix of extracellular polysaccharides (EPS), extracellular DNA, proteins and lipids, changing their structure and functional relationships (Stoodley et al. 2002). But bacterial biofilms can also exist in the air-liquid interface forming floating biofilms or pellicles. This interface provides access to oxygen and other gasses from the air, as well as nutrients from the liquid phase through opposing gradients (Armitano et al. 2014).

*Microcystis* colonies are extremely buoyant, commonly forming wind-blown scums. Their position relative to the surface can be achieved thanks to the presence of gas vesicles aggregations in the cytoplasm (Šmarda and Maršálek 2008), which allow them to regulate their vertical position in the water column and to form the colony in a suitable position to receive the right amount of light, oxygen, CO_2_ and nitrogen, which is necessary to build the protein vesicles (Wu et al. 2023). The EPS matrix contributes to buoyancy and has the same composition that has been described for pellicle-forming bacteria (Armitano et al. 2014), such as glucose, galactose, rhamnose, mannose or cellulose (Lei et al. 2007, 2009). This matrix creates a microenvironment called the phycosphere, where complex ecological interactions between phytoplankton and bacteria occur (Seymour et al. 2017).

Colony formation in *Microcystis* can be induced by abiotic factors causing stress, such as low temperature (15°C) and low light intensity (10 µmol photons m^−2^ s^−1^) (Yang et al. 2012, Li et al. 2013, Xu et al. 2016). In the presence of high concentration of calcium (Wang et al. 2011, Sato et al. 2017) and lead, the formation of colonies reaching more than 100 µm diameter can be induced and its EPS acts trapping the metal ions (Bi et al. 2013). As for bacterial biofilms, the ability of *Microcystis* to form colonies has also been linked to antibiotic resistance, since low concentrations of aminoglycoside antibiotics induced cell aggregation, suggesting a protective role for the EPS (Tan et al. 2018). Another characteristic shared by biofilms and *Microcystis* colonies is cellular motility. Genes encoding for type IV pili (e.g. *pilT*) have been found in *Microcystis aeruginosa* PCC 7806 (Nakasugi and Neilan 2005), which may indicate that cells can move by means of twitching motility during the initial arrangement of the cells inside the growing colony (Maier and Wong 2015). As the colony grows and the biofilm starts to mature, water channels develop and a differentiation in physiological processes among cells start to establish in response to conditions in their particular environments.

There is growing evidence relating colony size with the amount of microcystin they produce. For example, it has been shown that colonies in the size range from 60 to150 µm diameter are those producing higher amounts of microcystins compared to single cells or smaller colonies (Gan et al. 2012, Deus Álvarez et al. 2020). On the other hand, depletion of extracellular microcystin concentrations showed a decrease in colony size (Gan et al. 2012). Thus, the evidence suggests that released microcystins could act as an infochemical-related mechanism involved in the biofilm maintenance. However, if microcystins are involved in a QS-like mechanism remains uncertain.

Regarding QS, acylated homoserine lactones (AHLs) have been found in cultures of *M. aeruginosa* PCC-7820 (Zhai et al. 2012). Electron microscope photographs of *M. aeruginosa* supplemented with AHLs showed a shift from single free-living cells to a biofilm-like membrane. This suggests that QS might play an important role in the environmentally-driven morphological changes of *M. aeruginosa*, providing strong evidence that it regulates colony formation through a coordinated multicellular behaviour as that described for biofilms. This was confirmed more recently, when addition of several AHLs from Gram negative bacteria to cultures of *Microcystis* induced colony formation (Herrera and Echeverri 2021). The fact that AHLs belonging to several species were able to induce a response in *Microcystis* implies that the QS behaviour leading to colony formation could be triggered by members of the microbiome. Moreover, (Shi et al. 2022) showed that several transcripts for pathways involved in biofilm formation were enriched in the *Microcystis* colonial form compared to single cells. These transcripts belonged mainly to heterotrophic bacteria from the microbiota, meaning that QS in *Microcystis* is an ability conferred by the cyanobacterium and its microbiome acting cooperatively. This kind of multi-species, multicellular behaviour may have ecosystem-level effects on several processes, e.g. nutrient cycling, toxin biosynthesis, bloom stability, etc. (Van Le et al. 2022).

### The *Microcystis* holobiont

Current vision of organism´s evolution is increasingly incorporating the concept of holobiont, which recognizes the widespread occurrence of host-associated microbiomes and makes emphasis on the multispecies nature of host–microbiome assemblage (Bordenstein and Theis 2015). In the case of *Microcystis*, the colonial organism is in fact composed of a myriad of different bacterial species interacting and exchanging common goods (nutrients, gasses, carbon, genes) inside the mucilaginous envelope of the cyanobacterium, which confers it an extremely high ability to survive in different environmental conditions (Cook et al. 2020). Thus, it seems sound to conclude that the colonial organism we call *Microcystis* is in fact a holobiont. But, how is this prokaryotic holobiont formed?

It has been reported that the highly diverse microbiome of *Microcystis* colonies differs markedly from that present in single cells (Wu et al. 2019). Co-cultivation of axenic, single-celled cultures of *Microcystis* with heterotrophic bacteria isolated from *Microcystis* colonies stimulated cyanobacterial growth and induced the production of EPS, allowing to reconstitute colony formation (Reynolds 2007, Shen et al. 2011, Wang et al. 2016). Moreover, the existence of a metabolic interdependence between *Microcystis* and its microbiome has been proposed (Jackrel et al. 2019, Cook et al. 2020), suggesting that the ability to compete with other phytoplankton groups would not be determined by the toxin production but by genes from its microbiome (Schmidt et al. 2020). Therefore, there is evidence of a clear and strong relationship between the presence of an extracellular matrix and the recruitment of heterotrophic bacteria, which stimulate colonial growth through QS to form a three-dimensional structure where the exchange of common goods occurs. This constitutes a complex holobiont organism whose formation must have involved the establishment of a symbiotic relationship early in the evolution of the cyanobacterium. As a unicellular cyanobacteria, *Microcystis* can only achieve a multicellular stage through its relationship with the symbiotic partners. This hypothesis would also explain the reversion from colonies to single cells observed when isolating *Microcystis* from environmental samples (Wang et al. 2015), probably due to the several dilutions and washing steps that remove the attached bacteria.

### Conceptual model for colony formation in *Microcystis* holobiont

**Figure 1.**
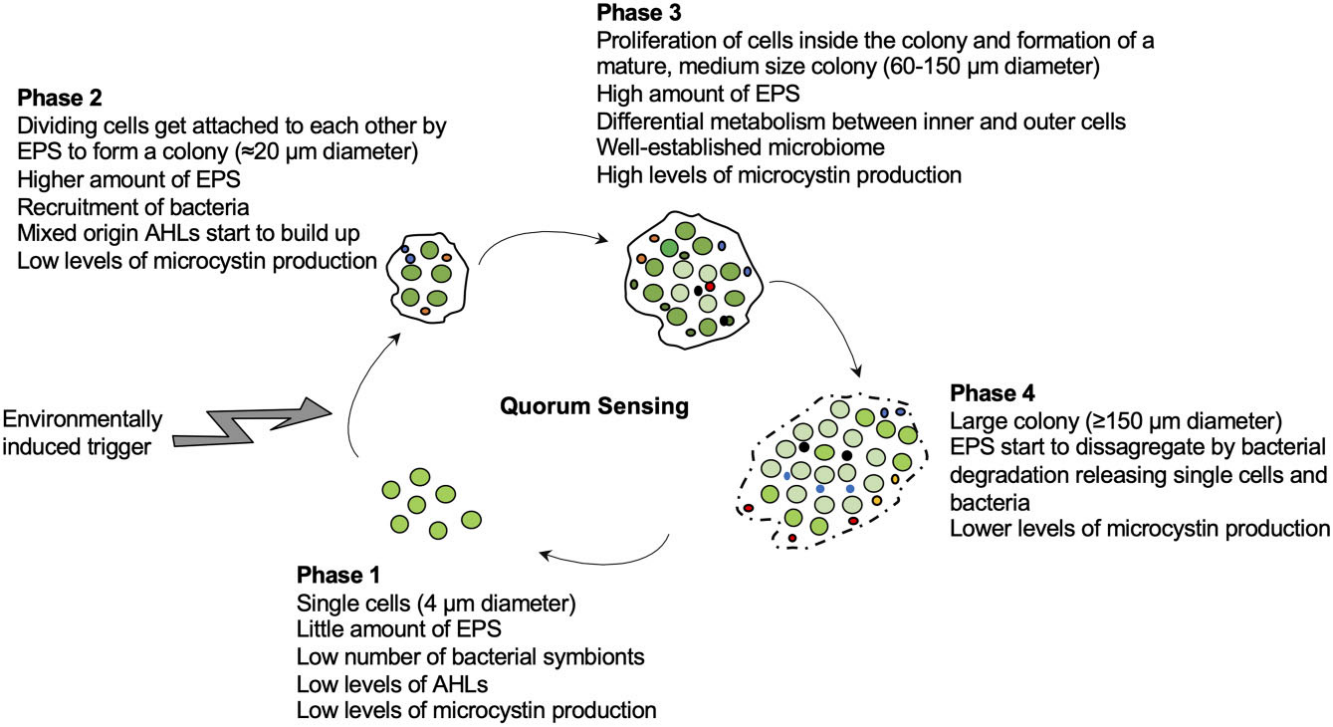
Proposal for the floating biofilm formation of *Microcystis*. Four phases can be distinguished during the development of a *Microcystis* biofilm according to its lifestyle (single celled vs. attached aggregate), EPS and microcystin production, presence of an established microbiome and autoinducers concentration (AHLs). Phase 1 is composed of single cells (4 µm diameter, green circles) having little amount of EPS mucilage, low levels of microcystin production and low levels of AHLs. Phase 2 starts with the initial attachment of dividing cells to each other to form a colony surrounded by a higher amount of EPS mucilage, cells probably mobilize inside the colony and they have low levels of microcystin synthesis while AHLs start to build up and other bacteria (smaller red, blue and black circles) start to be recluted and attached to the EPS. In Phase 3, the proliferation of bacterial cells inside the colony and their interactions with cyanobacterial cells allow the formation of a mature biofilm, with elevated amounts of EPS, high levels of microcystin production and clearly different metabolism between inner and outer cells. A microbiome is well established. The Phase 4 is characterized by large, amorphous colonies, low levels of microcystin production and disaggregation of the mucilage by bacterial degradation of the EPS (typically at the end of a bloom). We propose that the onset of a bloom will depend on abiotic and biotic conditions and on the phase of the *Microcystis* community, being more likely to develop a high biomass in a short time period during phase 3 (active cells, with high microcystin production rates).

The information gathered so far about colony formation in *Microcystis* spp. suggests that the mechanisms involved in this process are the same as those defined for biofilm formation in a number of bacterial species. *Microcystis* can switch from single cells to colonies organized into a coordinated functional community that is embedded in an EPS matrix teemed with a diversity of heterotrophic bacteria living mainly in a cooperative manner with the cyanobacterial cells (Fig. 1). The change from single cells to multicellular organization would be triggered by autoinducers molecules (e.g. AHLs) synthesized either by the cyanobacterium, by the microbiome, or both, in response to environmental cues (e.g. resource-rich conditions). As the population grows, the resources become less available and the AHLs upregulate a number of functional genes allowing the organisms to thrive under conditions that would not be favourable, such as nutrients or light shortage, oxidative stress, etc. The main components of the biofilm mucilage are EPS, DNA from lysed cells, proteins, lipids and heterotrophic bacteria that live embedded in this matrix. This bacterial community has a very constant structure, its functional relationships with the cyanobacterium are closely intertwined and involves the trade of different goods, allowing the holobiont to survive. The resulting multi-specific biofilm is not built from the attachment of the cells to an abiotic or biotic surface, but on the attachment of cells to each other to form a floating biofilm that thrives in a highly diverse array of environmental conditions.

### Future directions

Understanding the mechanism underlying the multispecific biofilm (colony) formation in *Microcystis* holobiont would help to unveil the role of the microbiome in the evolution and environmental performance of these organisms. This will be useful to determine not only the biotic or abiotic conditions triggering microcystin production, but also to uncover the role of microcystin in the holobiont ecology and, therefore, in blooms development. We expect that this kind of knowledge would improve current (and sometimes contradictory) models of growth, fitness, dispersal and decay of these cyanobacteria, contributing to water management and risk assessment.

## References

[bib1] Armitano J, Méjean V, Jourlin-Castelli C. Gram-negative bacteria can also form pellicles. Environ Microbiol Rep. 2014;6:534–44. 25756106 10.1111/1758-2229.12171

[bib2] Bi X, Zhang S, Dai W et al. Effects of lead (II) on the extracellular polysaccharide (EPS) production and colony formation of cultured Microcystis aeruginosa. Water Sci Technol. 2013;67:803–9. 23306258 10.2166/wst.2012.632

[bib3] Bordenstein SR, Theis KR. Host biology in light of the microbiome: ten principles of holobionts and hologenomes. PLoS Biol. 2015;13:e1002226. 26284777 10.1371/journal.pbio.1002226 PMC4540581

[bib4] Carrascal OMP, Tromas N, Terrat Y et al. Phylosymbiosis in the Microcystis microbiome. 2021.

[bib5] Cook KV, Li C, Cai H et al. The global Microcystis interactome. Limnol Oceanogr. 2020;65:S194–207. 32051648 10.1002/lno.11361 PMC7003799

[bib6] Dai R, Li Z, Yan F et al. Evaluation of changes in M. aeruginosa growth and microcystin production under phosphorus starvation via transcriptomic surveys. Sci Total Environ. 2023;893:164848. 37329914 10.1016/j.scitotenv.2023.164848

[bib7] Davis TW, Berry DL, Boyer GL et al. The effects of temperature and nutrients on the growth and dynamics of toxic and non-toxic strains of Microcystis during cyanobacteria blooms. Harmful Algae. 2009;8:715–25.

[bib8] Deus Álvarez S, Kruk C, de la Escalera GM et al. Morphology captures toxicity in Microcystis aeruginosa complex: evidence from a wide environmental gradient. Harmful Algae. 2020;97:101854. 32732048 10.1016/j.hal.2020.101854

[bib9] Dick GJ, Duhaime MB, Evans JT et al. The genetic and ecophysiological diversity of Microcystis. Environ Microbiol. 2021;23:7278–313. 10.1111/1462-2920.15615. 34056822

[bib10] Flemming HC, Wingender J. The biofilm matrix. Nat Rev Micro. 2010;8:623–33. 10.1038/nrmicro2415 20676145

[bib11] Gan N, Xiao Y, Zhu L et al. The role of microcystins in maintaining colonies of bloom-forming Microcystis spp. Environ Microbiol. 2012;14:730–42. 22040118 10.1111/j.1462-2920.2011.02624.x

[bib12] Herrera N, Echeverri F. Evidence of quorum sensing in Cyanobacteria by Homoserine Lactones: the origin of blooms. Water. 2021;13:1831. 10.3390/w13131831 (November 2022, date last accessed).

[bib13] Huisman J, Codd GA, Paerl HW et al. Cyanobacterial blooms. Nat Rev Micro. 2018;16:471–83. 10.1038/s41579-018-0040-1 29946124

[bib14] Jackrel SL, White JD, Evans JT et al. Genome evolution and host-microbiome shifts correspond with intraspecific niche divergence within harmful algal bloom-forming Microcystis aeruginosa. Mol Ecol. 2019;28:3994–4011. 31344288 10.1111/mec.15198

[bib15] Jankowiak JG, Gobler CJ. The composition and function of microbiomes within microcystis colonies are significantly different than native bacterial assemblages in two North American lakes. Front Microbiol. 2020;11. 10.3389/fmicb.2020.01016 (August 2021, date last accessed). PMC7270213 32547511

[bib16] Kruk C, Segura A, Piñeiro G et al. Rise of toxic cyanobacterial blooms is promoted by agricultural intensification in the basin of a large subtropical river of South America. Global Change Biol. 2023;29:1774–90., 10.1111/gcb.16587 36607161

[bib17] Lei LM, Song LR, Ou DY et al. Effects of nutrient conditions on exopolysaccharide production in water-bloom forming Cyanobacteria, Microcystis aeruginosa. Acta Sci Nat Univ Sunyatseni. 2007;46:84–7.

[bib18] Li M, Zhu W, Gao L et al. Seasonal variations of morphospecies composition and colony size of microcystis in a shallow hypertrophic lake (Lake Taihu, China). Fresenius Environ Bull. 2013;22:3474–83.

[bib19] Li P, Cai Y, Shi L et al. Microbial degradation and preliminary chemical characterization of Microcystis exopolysaccharides from a cyanobacterial water bloom of Lake Taihu. Int Rev Hydrobiol. 2009;94:645–55.

[bib20] Maier B, Wong GCL. How bacteria use type IV pili machinery on surfaces. Trends Microbiol. 2015;23:775–88. 26497940 10.1016/j.tim.2015.09.002

[bib21] Martin RM, Moniruzzaman M, Stark GF et al. Episodic decrease in temperature increases mcy gene transcription and cellular microcystin in continuous cultures of Microcystis aeruginosa PCC 7806. Front Microbiol. 2020;11:601864. 33343544 10.3389/fmicb.2020.601864 PMC7744600

[bib22] Nakasugi K, Neilan BA. Identification of pilus-like structures and genes in Microcystis aeruginosa PCC7806. Appl Environ Microb. 2005;71:7621–5. 10.1128/AEM.71.11.7621-7625.2005 PMC1287722 16269818

[bib23] Nickel JC, Ruseska I, Wright JB et al. Tobramycin resistance of Pseudomonas aeruginosa cells growing as a biofilm on urinary catheter material. Antimicrob Agents Chemother. 1985;27:619–24. 3923925 10.1128/aac.27.4.619 PMC180108

[bib25] Paerl HW, Huisman J. Blooms like it hot. Science. 2008;320:57–8. 18388279 10.1126/science.1155398

[bib24] Paerl HW . Nuisance phytoplankton blooms in coastal, estuarine, and inland waters. Limnol Oceanogr. 1988;33:823–43.

[bib26] Petrova OE, Sauer K. Escaping the biofilm in more than one way: desorption, detachment or dispersion. Curr Opin Microbiol. 2016;30:67–78. 26826978 10.1016/j.mib.2016.01.004 PMC4821722

[bib27] Pimentel JS, Giani A. Microcystin production and regulation under nutrient stress conditions in toxic microcystis strains. Appl Environ Microbiol. 2014;80:5836–43. 25038094 10.1128/AEM.01009-14 PMC4178597

[bib29] Reynolds CS, Jaworski GHM, Cmiech HA et al. On the annual cycle of the blue-green alga Microcystis aeruginosa Kütz. Emend. Elenkin. Philos Trans R Soc London B, Biol Sci. 1981;293:419–77.

[bib28] Reynolds CS . Variability in the provision and function of mucilage in phytoplankton: facultative responses to the environment. Hydrobiologia. 2007;578:37–45.

[bib30] Sato M, Amano Y, Machida M et al. Colony formation of highly dispersed Microcystis aeruginosa by controlling extracellular polysaccharides and calcium ion concentrations in aquatic solution. Limnology. 2017;18:111–9.

[bib31] Sauer K, Stoodley P, Goeres DM et al. The biofilm life cycle: expanding the conceptual model of biofilm formation. Nat Rev Micro. 2022;20:608–20. 10.1038/s41579-022-00767-0 PMC9841534 35922483

[bib32] Schmidt KC, Jackrel SL, Smith DJ et al. Genotype and host microbiome alter competitive interactions between Microcystis aeruginosa and Chlorella sorokiniana. Harmful Algae. 2020;99:101939. 33218432 10.1016/j.hal.2020.101939

[bib33] Seymour JR, Amin SA, Raina J-B et al. Zooming in on the phycosphere: the ecological interface for phytoplankton–bacteria relationships. Nat Microbiol. 2017;2:17065. 28555622 10.1038/nmicrobiol.2017.65

[bib34] Shen H, Niu Y, Xie P et al. Morphological and physiological changes in Microcystis aeruginosa as a result of interactions with heterotrophic bacteria. Freshw Biol. 2011;56:1065–80.

[bib35] Shi L, Cai Y, Gao S et al. Gene expression in the microbial consortia of colonial Microcystis aeruginosa-a potential buoyant particulate biofilm. Environ Microbiol. 2022;24:4931–45. 35837847 10.1111/1462-2920.16133

[bib36] Šmarda J, Maršálek B. Microcystis aeruginosa (Cyanobacteria): ultrastructure in a pelagic and in a benthic ecosystem. Arch Hydrobiol Suppl Algol Stud. 2008;126:73–86.

[bib37] Stal LJ . Cyanobacterial mats and stromatolites. Ecology of Cyanobacteria II. Dordrecht: Springer, 2012, 65–125.

[bib38] Stewart PS, Franklin MJ. Physiological heterogeneity in biofilms. Nat Rev Micro. 2008;6:199–210. 10.1038/nrmicro1838 18264116

[bib39] Stoodley P, Sauer K, Davies DG et al. Biofilms as complex differentiated communities. Annu Rev Microbiol. 2002;56:187–209. 12142477 10.1146/annurev.micro.56.012302.160705

[bib40] Tan L-R, Xia P-F, Zeng RJ et al. Low-level concentrations of aminoglycoside antibiotics induce the aggregation of cyanobacteria. Environ Sci Pollut Res. 2018;25:17128–36. 10.1007/s11356-018-1894-5 29644613

[bib41] Van de Waal DB, Verspagen JMH, Finke JF et al. Reversal in competitive dominance of a toxic versus non-toxic cyanobacterium in response to rising CO2. ISME J. 2011;5:1438–50. 21390081 10.1038/ismej.2011.28 PMC3160686

[bib42] Van Le V, Srivastava A, Ko S-R et al. Microcystis colony formation: extracellular polymeric substance, associated microorganisms, and its application. Bioresour Technol. 2022;360:127610. 35840029 10.1016/j.biortech.2022.127610

[bib43] Wang W, Shen H, Shi P et al. Experimental evidence for the role of heterotrophic bacteria in the formation of Microcystis colonies. J Appl Phycol. 2016;28:1111–23.

[bib44] Wang W, Zhang Y, Shen H et al. Changes in the bacterial community and extracellular compounds associated with the disaggregation of Microcystis colonies. Biochem Syst Ecol. 2015;61:62–6.

[bib45] Wang Y-W, Zhao J, Li J-H et al. Effects of calcium levels on colonial aggregation and buoyancy of Microcystis aeruginosa. Curr Microbiol. 2011;62:679–83. 20872220 10.1007/s00284-010-9762-7

[bib46] Wu Q, Zhang Y, Li Y et al. Comparison of community composition between Microcystis colony-attached and free-living bacteria, and among bacteria attached with Microcystis colonies of various sizes in culture. Aquat Ecol. 2019;53:465–81.

[bib47] Wu T, Wang C, Cao J et al. Coupling of light and nutrients affects Microcystis gas vesicle content at different depths. J Plankton Res. 2023;45:467–77.

[bib48] Xu F, Zhu W, Xiao M et al. Interspecific variation in extracellular polysaccharide content and colony formation of Microcystis spp. cultured under different light intensities and temperatures. J Appl Phycol. 2016;28:1533–41.

[bib49] Yang X, Bi Y, Ma X et al. Transcriptomic analysis dissects the regulatory strategy of toxic cyanobacterium Microcystis aeruginosa under differential nitrogen forms. J Hazard Mater. 2022;428:128276. 35051775 10.1016/j.jhazmat.2022.128276

[bib50] Yang Z, Geng L, Wang W et al. Combined effects of temperature, light intensity, and nitrogen concentration on the growth and polysaccharide content of Microcystis aeruginosa in batch culture. Biochem Syst Ecol. 2012;41:130–5.

[bib51] Yang Z, Kong F, Shi X et al. Changes in the morphology and polysaccharide content of Microcystis aeruginosa (Cyanobacteria) during flagellate grazing. J Phycol. 2008;44:716–20. 27041430 10.1111/j.1529-8817.2008.00502.x

[bib52] Yin L, Xu L, Shi K et al. Physiology, microcystin production, and transcriptomic responses of Microcystis aeruginosa exposed to calcium and magnesium. Sci Total Environ. 2024;913:169786. 38181954 10.1016/j.scitotenv.2023.169786

[bib53] Zhai C, Zhang P, Shen F et al. Does Microcystis aeruginosa have quorum sensing?. FEMS Microbiol Lett. 2012;336:38–44. 22861498 10.1111/j.1574-6968.2012.02650.x

[bib54] Zhou Y, Li X, Xia Q et al. Transcriptomic survey on the microcystins production and growth of Microcystis aeruginosa under nitrogen starvation. Sci Total Environ. 2020;700:134501. 31689655 10.1016/j.scitotenv.2019.134501

